# Does PGE_1_ Vasodilator Prevent Orthopaedic Implant-Related Infection in Diabetes? Preliminary Results in a Mouse Model

**DOI:** 10.1371/journal.pone.0094758

**Published:** 2014-04-09

**Authors:** Arianna B. Lovati, Carlo L. Romanò, Lorenzo Monti, Christian Vassena, Sara Previdi, Lorenzo Drago

**Affiliations:** 1 Cell and Tissue Engineering Laboratory, IRCCS Galeazzi Orthopaedic Institute, Milan, Italy; 2 Dipartimento di Chirurgia Ricostruttiva e delle Infezioni Osteo-articolari, IRCCS Galeazzi Orthopaedic Institute, Milan, Italy; 3 Laboratory of Clinical Chemistry and Microbiology, IRCCS Galeazzi Orthopaedic Institute, Milan, Italy; 4 Laboratory of Cancer Cachexia AIRC Start-Up, Oncology Department, Istituto di Ricerche Farmacologiche Mario Negri, Milan, Italy; 5 Department of Biomedical Science for Health, University of Milan, Milan, Italy; University of Rochester, United States of America

## Abstract

**Background:**

Implant-related infections are characterized by bacterial colonization and biofilm formation on the prosthesis. Diabetes represents one of the risk factors that increase the chances of prosthetic infections because of related severe peripheral vascular disease. Vasodilatation can be a therapeutic option to overcome diabetic vascular damages and increase the local blood supply. In this study, the effect of a PGE_1_ vasodilator on the incidence of surgical infections in diabetic mice was investigated.

**Methodology:**

A S. aureus implant-related infection was induced in femurs of diabetic mice, then differently treated with a third generation cephalosporin alone or associated with a PGE_1_ vasodilator. Variations in mouse body weight were evaluated as index of animal welfare. The femurs were harvested after 28 days and underwent both qualitative and quantitative analysis as micro-CT, histological and microbiological analyses.

**Results:**

The analysis performed in this study demonstrated the increased host response to implant-related infection in diabetic mice treated with the combination of a PGE_1_ and antibiotic. In this group, restrained signs of infections were identified by micro-CT and histological analysis. On the other hand, the diabetic mice treated with the antibiotic alone showed a severe infection and inability to successfully respond to the standard antimicrobial treatment.

**Conclusions:**

The present study revealed interesting preliminary results in the use of a drug combination of antibiotic and vasodilator to prevent implant-related Staphylococcus aureus infections in a diabetic mouse model.

## Introduction

Implant-related infection has been recently reported as, respectively, the first and the third reason for failure of knee and hip prosthesis in the U.S. [1], [2]. Infection rates after revision surgery are considerably higher (5–40%) than after primary replacement [3].

Implant-related infections represent the 65% of orthopaedic infections and they are characterized by bacterial colonization and biofilm formation on the prosthetic implant and within the contiguous tissues [4]. Bacteria within biofilm, in particular *Staphylococcus aureus*, are extremely resistant to antibiotics and persistent infections arise despite proper therapies [5]. Treatments usually involve debridement procedures, surgical revisions and long term antibiotic therapy. Nevertheless, some infections are not entirely eradicated and lead to implant failure or loss [6].

Patient co-morbidities - diabetes, obesity, immunodeficiency and vascular diseases – represent risk factors that increase the chances of implant-related infections. In particular, diabetes alters the tissue healing and induces a high susceptibility to infections with risk of mortality [7]. Diabetes results in several disorders such as peripheral neuropathy, vasculopathy and ischemia due to the compromised granulocyte adherence [8], [9]. Neuropathy and angiopathy play a primary role in the development of infections in diabetic patients, in particular, the implant-site districts of these patients respond inadequately to pharmacological treatments [10], [11]. Moreover, the decrease of blood supply increases tissue necrosis near the implant, reduces the healing process, and contributes to the development of osteomyelitis [12]. Frequently, these events lead to the implant loss and to revision surgeries with high costs. Thus, the reasons for failure of antibiotic treatments may be due to the severe peripheral vascular disease and unsuccessful revascularization. In fact, the reduced peripheral blood flow in diabetics has been demonstrated to impede the systemic antibiotics to reach superficial wound and ulcers, thus limiting the effective control of continued tissue infection by bacteria [13]. A successful management of peri-prosthetic infections in diabetics is strictly based on their prevention and novel therapeutic approaches. The stimulation of local blood supply could be a therapeutic option to balance the vascular damages [14], [15]. Prostaglandin E_1_ (PGE_1_) is a powerful vasodilator able to increase the peripheral blood perfusion by enhancing the endothelial function [16], [17]. PGE_1_ is already used for the treatment of chronic occlusive arterial disease [18] as either vasodilator or inhibitor of platelet aggregation [15], [17], [19], [20]. PGE_1_ plays a role to increase skin and muscle blood flow [21] as well as to generate new blood capillaries in ischemic skeletal muscles [22]. Moreover, the reduction of infections demonstrates the efficacy of PGE_1_ in patients with prior irradiation after laryngeal surgeries [23]. Others demonstrated that the treatment of wounds with vasodilators in rats increased the local blood flow and antibiotic delivery to the site of injury [13]. Therefore, it is hypothesized that PGE_1_ administration might decrease the incidence of surgical infections in diabetic patients. However, the role of PGE_1_ in implant-related infections has not yet been evaluated.

In the present study, we investigated the effects of a PGE_1_ on implant-related infections in a diabetic mouse model, as already described in our previous work [24]. To verify our hypothesis, we compared data obtained from mice treated with a cephalosporin or with the association of the cephalosporin and a PGE_1_ vasodilator.

## Materials and Methods

### Ethics Statement

The Mario Negri Institute for Pharmacological Research (IRFMN) Animal Care and Use Committee (IACUC) approved the whole study (Permit N. 43_2013-B). Animals and their care were handled in compliance with institutional guidelines as defined in national (Law 116/92, Authorization n.19/2008-A issued March 6, 2008, by the Italian Ministry of Health) and international laws and policies (EEC Council Directive 86/609, OJ L 358. 1, December 12, 1987; Standards for the Care and Use of Laboratory Animals - UCLA, U. S. National Research Council, Statement of Compliance A5023-01, November 6, 1998). The animals were housed at the Institute's Animal Care Facilities that meet international standards; they were regularly checked by a certified veterinarian responsible for health monitoring, animal welfare supervision, experimental protocols and procedure revision.

### Experimental design

The effects of the association of a PGE_1_ vasodilator and a cephalosporin were tested on a previously validated diabetic mouse model of staphylococcal orthopaedic implant-related infection [24].

To this aim, NOD/ShiLtJ mice were assigned to one of three experimental groups (n = 8 animals in each group):

Group I Sham control (3 μl PBS + Cephalosporin)
Group II Antibiotic treatment (*S. aureus* 10^3^ CFU/3 μl + Cephalosporin)
Group III Combined treatment (*S. aureus* 10^3^ CFU/3 μl + Cephalosporin + PGE_1_ vasodilator)

### Preparation of *S. aureus* for inoculation into the joint space

*S. aureus* strain ATCC 25923 was used in this study as described in our recent study [24]. Briefly, bacteria were cultured at 37°C overnight onto Mannitol Salt Agar (BioMerieux, France) and incubated into Brain Heart Infusion Broth (BioMerieux) at 37°C for 16 hours. The bacterial suspension was suspended in PBS to obtain a 0.5 McFarland turbidity (equal to about 1×10^8^ CFU/mL), then serially diluted with sterile saline solution and counts were performed to check for bacterial inoculum used for the experiments.

### *In vivo* surgical procedures

Twenty-four female NOD/ShiLtJ type I diabetic 14 week old mice (mean body weight 23.3±1.3 g) (Jackson Laboratory) were used for this experiment. Blood glucose levels for diabetes were tested in the NOD/ShiLtJ mice directly by the provider before delivery. The mice were maintained under specific pathogen-free conditions and food was provided *ad libitum*. All procedures on the animals were performed under a laminar flow hood. The implantation of the intramedullary nail was performed as previously described [24], [25] and maintained *in situ* for 28 days. A bacterial suspension of about 1×10^3^ CFU/mouse was injected in group II and III into the femoral canal after implantation according to the literature [25], [26]. In the sham controls (group I), sterile PBS was injected as described above. Immediately after surgery, all animals received a one-shot injection of carprofen 5 mg/kg SC (Rimadyl, Pfizer, Italy) and ceftriaxone 60 mg/kg IM (Rocephin, Roche, Italy). The cephalosporin bactericidal effect on *S. aureus* strain ATCC 25923 was previously tested in vitro. Additionally, group III was treated intravenously with a PGE_1_ vasodilator at a dosage of 10 μg/kg (Prostavasin, Schwarz Pharma, Italy).

The animals were housed in separate cages for 24 h, then grouped four per cage, and daily clinically monitored. Pain was controlled with buprenorphine (0.1 mg/kg SC). After 4 weeks, the mice were euthanized by CO_2_ inhalation to perform the investigations on the harvested samples.

### Blood collection and analysis

To determine the total white blood cells (WBC) count, blood samples were collected from the animals' facial vein (n = 24) on day 0 and from the left ventricle immediately after sacrifice (day 28) as described by Lovati et al. 2013 [24]. EDTA anti-coagulated blood samples were used to obtain values of total WBC with an automatic cell counter for human use (Sysmex XT-1800, Dasit).

### Micro-CT imaging and data analysis

To evaluate bone reaction, micro-CT analysis (n = 5 per group) were performed by two independent examiners on explanted femurs with an Explore Locus micro-CT scanner (GE Healthcare, London, Ontario, Canada), without using contrast agents. Protocols and procedures of micro-CT scan acquisitions were already described by Lovati et al. 2013 [24]. The images from each sample were binarized at identical thresholds to allow for unbiased identification of bone damage and osteolysis.

The image analysis was designed on a volume of interest (VOI) to evaluate the outer bone volume of the femur to measure any anatomical changes. Bone mineral density (BMD) was measured after calibration using a phantom placed in the field of view of the scanned specimens. The BMD (mg/cc) was measured on the bone volume designed on the femoral bone by the Micro View image viewer (version 2.1.2; GE Healthcare). BMD data were then normalized on the baseline BMD obtained in the control group I.

### Histological analysis

Femoral specimens (n = 4 per group) were fixed in 10% formalin overnight, then decalcified in Mielodec (Bio-Optica, Milan, Italy), dehydrated, the metallic implants were removed, samples were embedded in paraffin, and cut into 5 μm sagittal sections. After deparaffinization, the slides were stained with Haematoxylin and Eosin (H&E) and Gram staining to assess the presence of bacteria, finally analyzed by three independent examiners using an Olympus IX71 light microscope. The inflammatory response and infection were both evaluated trough the periosteal reaction, cortical bone and medullary canal changes according to the grading score (0 to 3) described in our previous study [24]. Briefly, the periosteum was analyzed for absence or presence of reaction; the cortex was mainly analyzed for absence or presence of polymorpho-nucleated cells, osteoclasts and bone resorption; and the medullary canal for absence or presence of polymorpho-nucleated cells and micro- and macroabscesses.

### Microbiological analysis

To quantify bacteria within the explanted samples (n = 4 per group), serial dilutions from sonicated fluids were plated onto Mueller-Hinton agar plates (Biomerieux, Marcy l'Etoile, France) and incubated for 16 h at 37°C. Briefly, sterile container containing explanted samples was filled with 1 ml of sterile saline and sonicated in an ultrasound bath (VWR, Milan, Italy) for 5 min with a frequency of 30 kHz and a power output of 300 W at room temperature. All samples were assayed by serial 10-fold dilution in sterile saline solution and then plated on solid growth medium. After incubation of the plates at 37°C for 16 h, colonies of Gram-positive catalase positive cocci, resembling those of *S. aureus*, were tested for coagulase activity with Coagulase Plasma (Remel Europe Ltd. Dartford, UK) and identified by means of API Staph assay (BioMerieux, Mercy L'Etoile, France). Positivity to catalase test consists in the development of gaseous oxygen when the colony was put in contact with oxygenate water. Coagulase test results positive when *S.aureus* colonies in contact with coagulase plasma, form a visible clot after incubation for 4–6 h at 37°C. Colonies identified as *S. aureus* grown on Mueller-Hinton agar plates were then counted. The detection limit (L. o. D) was ≤1.300 (Log CFU)/g of bone.

### Statistical analysis

Comparisons between groups were analyzed with one-way analysis of variance (ANOVA) (Instat 2.0; Graphpad Software, San Diego, CA). Comparisons between groups and time points were analyzed with two-way ANOVA. When significant differences were detected, post hoc comparisons of means were performed using Bonferroni's procedure. Comparison between two groups was analyzed with unpaired t-test. All data are expressed as means ± standard error (SEM). Values of *P*<0.05 were considered statistically significant.

## Results

### Gross appearance and clinical data

The NOD/ShiLtJ mice displayed plasma glucose levels higher than 130 mg/dl at 14 weeks of age.

Groups I and III showed a slight lameness that solved within few days after implantation. On the contrary, most of the animals of group II, except one, developed subcutaneous swelling or abscesses and a marked lameness persist until the day of explantation. After two weeks of implantation, one mouse of group II was euthanized because of the poor conditions and it was replaced by another animal. Four weeks after implantation, all mice were sacrificed and femurs were explanted. The gross appearance examination confirmed the clinical data reported above. Groups I and III did not show macroscopic signs of infection, while group II showed macroabscesses of the joint soft tissues and lymph node enlargement. Signs of infection in group II were related with a consistent loss of body weight by day 7 post-infection when compared with group I, which improved the body weight over time. The mice of group III showed a restricted body weight loss either at day 7 or at day 14 post-infection compared with group II. In group III, the body weight increased starting from day 14 and recovered better than group II at 28 days post-infection.

**Figure 1:**
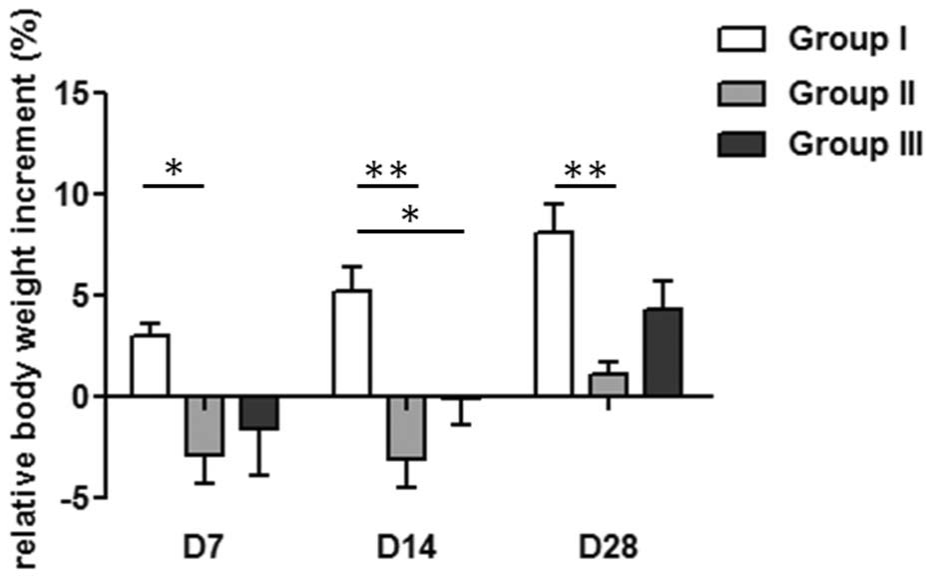
Relative changes in body weight. The histogram shows the relative changes in body weight in the experimental groups over time. A significant weight loss was measured in group II versus the control group I over time. Group III showed a significant body weight loss at day 14 after implantation compared to group I. Group III recovered body weight starting from the second week after surgery (two-way ANOVA, **P<0.01, *P<0.05; n = 8).

The histogram in Fig. 1 reports the percentage changes in body weight versus baseline (day of inoculation/implantation).

### Blood analysis

After 28 days, no statistical difference was calculated among the experimental groups. A mild WBC increase was measured in group II (3.13%) compared to group I (1.96%) and group III (2.29%). In particular, no effects of the PGE_1_ administration on the WBC count were detected in group III except a lower WBC decrease in group III compared with group II.

### Micro-CT imaging analysis

**Figure 2:**
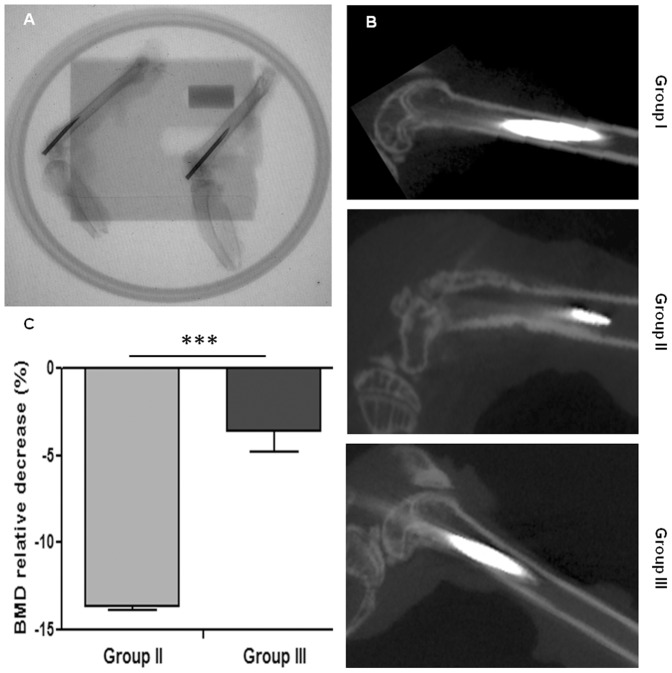
Representative micro-CT images and bone mineral density (BMD). A) Representative fluoroscopic image attests the correct placement of the implant within the femoral canal and the phantom calibration placed in the field of view; B) Magnified representative micro-CT images of the femurs containing metallic implants in transversal views in all the experimental groups. The images of groups I and III show an intact cortical bone profile of the whole femur. The image of group II shows a diffuse cortical and endosteal bone loss as signs of osteomyelitis; C) The histogram shows a high statistically significant difference in the BMD relative decrease of group II versus group III (unpaired t-test, ***P<0.0001; n = 5).

Micro-CT fluoroscopic examination confirmed the correct placement of the intramedullary implant within the femoral canal in all the experimental groups (Fig. 2A).

The qualitative micro-CT analysis showed no damages in the cortical and endosteal bone either along the diaphysis or femoral condyles in groups I and III. In group II, the *S. aureus* infection established a diffuse bone loss of the femoral metaphysis and diaphysis associated with the disruption of the endosteal bone, the decrease of the cortical bone thickness and the enlargement of the femoral canal (Fig. 2B). The BMD analysis was performed on values of groups II and III normalized on the BMD mean of group I, as sham control. Group II displayed a statistically significant decrease in BMD compared with group III, as shown in the histogram of Fig. 2C.

### Histological analysis of bones and joints

**Figure 3:**
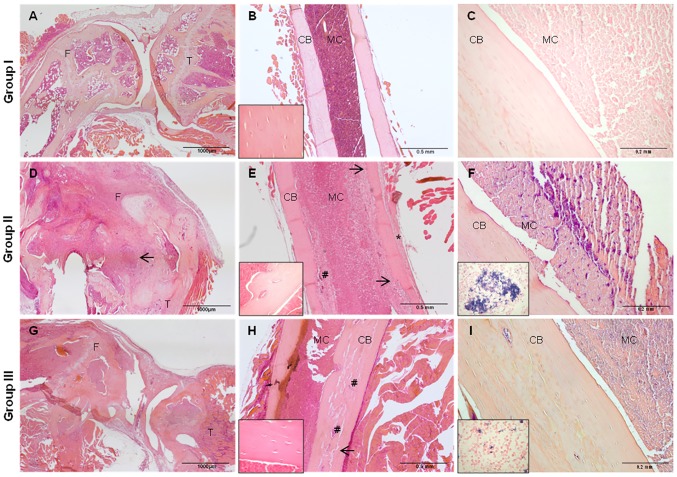
Histology of the femurs in the experimental groups (n = 4). Figures represent H&E staining in the left and middle panels and Gram-positive staining in the right panel. Legend: femur (F), tibia (T), cortical bone (CB), and medullary canal (MC). Group I - A) Normal aspect of the knee joint and absence of signs of infection (Magnification 2X, scale bar 1 mm); B) Absence of inflammatory cells in the medullary canal, of bone resorption or periosteal reaction (Magnification 4X, scale bar 0.5 mm) and presence of osteocytes within cortical bone lacunae (small box, Magnification 1000X); C) Absence of Gram-positive bacteria aggregates (Magnification 10X, scale bar 0.2 mm). Group II – D) Abscesses in the knee joint (black arrow) and in the medullary canal (Magnification 2X, scale bar 1 mm); E) Endosteal bone resorption (#), active osteoclasts (black arrows), marked periosteal reaction (*) (Magnification 4X, scale bar 0.5 mm) and diffuse enlargement of the medullary canal with osteoclastic resorption in the endosteal side (small box, Magnification 20X, scale bar 0.1 mm); F) Presence of numerous Gram-positive bacteria aggregates (Magnification 10X, scale bar 0.2 mm; small box, Magnification 1000X). Group III - G) Irregular surface of the articular cartilage of the knee joint and mild inflammatory changes of bone and joint (Magnification 2X, scale bar 1 mm); H) Diffuse increase of the vascular network and bone vessel enlargement (#) and areas of bone remodeling (black arrow) (Magnification 4X, scale bar 0.5 mm); large osteocytes embedded in cortical bone lacunae (small box, Magnification 1000X); I) Mild presence of dispersed Gram-positive bacteria within the medullary canal (Magnification 10X, scale bar 0.1 mm; small box, Magnification 1000X).

Group I explants showed a normal aspect of the knee joint, absence of any signs of infection (Fig. 3A) and normal sized femoral canal (Fig. 3B). No bone resorption or periosteal reactions were present in these samples, the cortical bone of the diaphysis was unaffected and osteocytes appeared small with dense flattened nuclei (Fig. 3B small box). No bacteria were identified in Gram-positive staining (Fig. 3C).

In contrast, in group II, joint and bone were affected by moderate to severe inflammatory changes multifocally extending to surrounding soft tissues (muscles, tendons and ligaments). Severe chronic neutrophilic osteomyelitis and arthrosynovitis were found with multifocal abscesses and pyogranulomas with intralesional bacteria (Fig. 3D). Marked bone resorption of cortical bone and periosteal reaction were present together with irregularities of the bone surface facing the bone marrow cavity and presence of osteoclasts in the endosteal side (Fig. 3E). Numerous intralesional aggregates of Gram-positive bacteria (cocci) were detected in the medullary canal as well as in the joint space and within the muscle fibers (Fig. 3F).

In group III, a partially irregular surface of the articular cartilage of the knee joint was detected together with mild inflammatory changes of bone and joint when compared to group II (Fig. 3G). Diaphysis cortical bone was irregularly thickened and had moderate to marked signs of bone remodeling (cement lines, new bone deposition) and overall appeared less dense with multifocal blood vessels, areas of woven bone and larger osteocytes compared to unaffected cortical bone of group I (Fig. 3H). Overall, dispersed Gram-positive bacteria were present in a smaller amount compared to group II (Fig. 3I).

**Figure 4:**
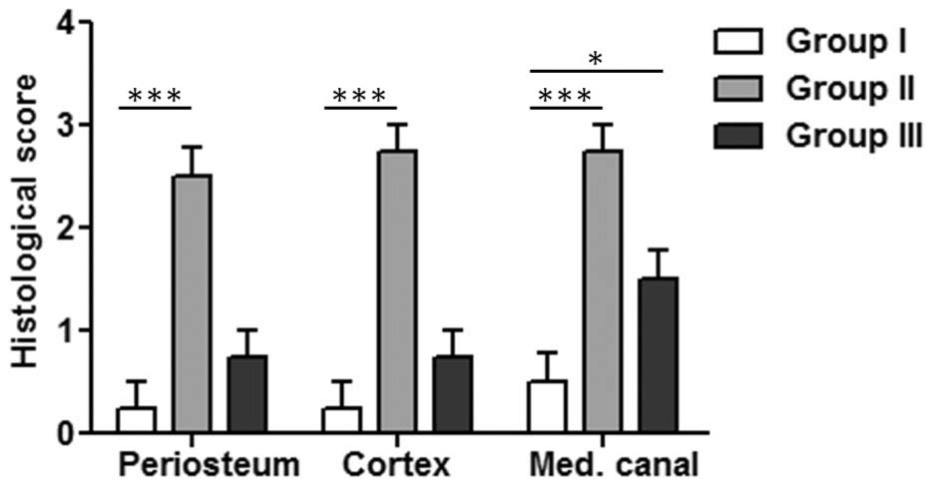
Histological grading score histogram. The histogram shows a high statistically significant difference in the histological grading score in periosteum, cortex and medullary canal of group I versus group II and a difference in the score of the medullary canal of group I versus group III (two-way ANOVA, ***P<0.001; *P<0.05; n = 4).

The histological grading score showed a higher significant difference in group I compared to group II for all the analyzed regions, and a significant difference was identified in the medullary canal of group I compared to group III, as reported in the histogram of Fig. 4.

### Microbiological analysis

**Figure 5:**
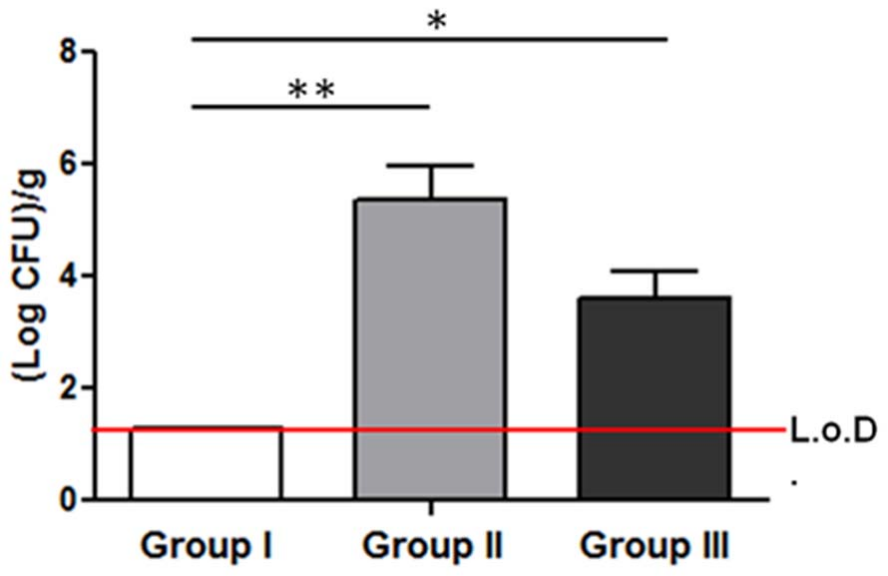
Bacterial load in bones of all the experimental groups. No colonies were detected in group I, the sham control (L.o.D.  =  limit of detection). With an infecting dose of 1×10^3^ CFU/mouse, a mean of 5.3±1.2 (Log CFU)/g of bone was found in the explants of group II, with a statistical difference in respect to group I. A mean of 3.6 ± 0.9 (Log CFU)/g of bone was found in the explants of group III, with a statistical difference in respect to group I (one-way ANOVA, *P<0.05; **P<0.01; n = 4).

After sonication, no bacterial growth was observed in group I (≤1.3 Log CFU/g bone). By contrast, great amounts of *S. aureus* bacteria were recovered in the samples of group II with a mean of 5.3±1.2 (Log CFU)/g bone. A restricted amount of bacteria was also measured in the samples of group III with a mean of 3.6±0.9 (Log CFU)/g bone. The histogram in Fig. 5 compares bacterial counts in all the experimental groups. A statistical difference was calculated between either group II or III compared with group I. Despite no statistically differences appeared between group II and III, the bacterial growth was lower in group III that received the association of antibiotic and vasodilator.

## Discussion

In diabetic patients, the peripheral blood supply is impaired due to various degree of angiopathy and microneurovascular dysfunction [27]. The reduced tissue perfusion may compromise the delivery of drugs, in particular, in the lower limbs [28], [29]. This impaired vascularization delays the tissue healing after surgery and increases the rate of implant-related infection despite aggressive antibiotic therapies [30]. Thus, the priority is to control the risk of infection by increasing both the vascular supply and the efficacy of antibiotics.

In this study, we found that the combination of a third generation cephalosporin with a PGE_1_ vasodilator improved the host response to the implant-related *S. aureus* infection in the diabetic mouse model, the latter already described in our previous study [24]. Many studies described the properties of PGE_1_ to maintain the blood perfusion, increase angiogenesis and inhibit platelet aggregation [16], [31], [32], [33]. On this basis, we hypothesized that the administration of a PGE_1_ might enhance the antibiotic delivery in the lower extremities and decrease the bacterial colonization of the infected site. In the present study, the intravenous administration of PGE_1_ has been chosen to avoid any potential systemic side effects as also suggested by others [22].

The change in body weight is an index of animal welfare, correlates with behavior in feeding and presence/absence of diseases [34]. The high statistical difference between group I and group II assesses the poor condition of the group II treated with the antibiotic alone, which showed an abnormal behavior in food intake. Differently, group III, treated with the association of the antibiotic and vasodilator, was able to regain body weight quicker and better respect to group II, and it showed no differences when compared to group I after 28 days from surgery. As reported elsewhere in diabetic patients, both the impaired bactericidal activity of polymorphonuclear cells [35]–[38] and the delayed hypersensitivity reaction of T-cell function [39], [40] explain the poor response to infection of group II, which showed a very low body weight increase over time. Differently, in group III, the quick body weight regain might be related to the efficacy of PGE_1_ in promoting the T-cell proliferation and oxygenation, as well as the response to infection and inflammation, as demonstrated also by others [41], [42]. Furthermore, PGE_1_ vasodilators have a direct cytoprotective effect by suppressing the production of proinflammatory cytokines [43], [44], thus PGE_1_ indirectly attenuate the cytotoxic effects of inflammation and improve the host defense [45]. To strongly support the PGE_1_ modulatory effect on inflammation, the serum concentration of proinflammatory cytokines should be investigated (e.g. C reactive protein). Our study lacks of this analysis because of the not correctly established values in mouse serum [24], [46].

Despite no statistical difference exits in WBC count among groups, the relative increase in total WBC count was lower in group III respect to group II. An increase in WBC count was expected because PGE_1_ commonly inhibits the activation and adhesion of polymorphonuclear neutrophils. However, PGE_1_ suppresses the release of proinflammatory cytokines from activated mononuclear cells, cytokines that can cause neutrophilia as demonstrated in animals [47] and humans [48]. PGE_1_ has also an inhibitory effect on granulocyte proliferation [49] and may be the explanation for our observation.

Ischemia and poor blood supply enhance the bone susceptibility to microbial invasion. As the infection reaches the medullary canal, the pressure increases and causes the bacterial extension into the cortex by bone canals widening into the periosteum and adjacent soft tissues [50]. In our study, micro-CT detected clear changes within the cortical bone due to the infection in group II, confirming the development of a chronic osteomyelitis. In contrast, group III showed no signs of osteomyelitis and a lower decrease in BMD compared to group II. These data support the efficacy of the PGE_1_ treatment to limit the bacterial extension within the bone tissue. Moreover, the higher BMD values in group III confirm the PGE_1_-mediated anabolic osteogenic response and bone remodeling, as also demonstrated in other studies [51], [52], [53].

Histology confirmed the micro-CT results showing evident signs of infection in group II as compared to the other groups. In particular, endosteal and intracortical resorption by osteoclasts, inflammatory cell infiltration, periosteal reaction and bacteria in the site of implantation in group II were present, as typical signs of infection as also described in different species by others [38], [54], [55]. Differently, both in group I and III no or mild signs of chronic osteomyelitis were detected, respectively, together with normal sized medullary canal and the presence of active osteoblats. Moreover, the increased cortical porosity and the bone remodeling in group III supported the hypothesis that PGE_1_ stimulates bone anabolism and subperiosteal bone formation thanks to osteoblast recruitment, as also described by others [51], [56], [57]. These observations are also consistent with observations following a single systemic administration of PGE_1_ in rats over a 4-week period [58], [59] and dogs [60]. In further studies, specific evaluations of vascular network could be useful to confirm the angiogenic and vasodilation effect of PGE_1_ on bone.

The microbiological analyses demonstrated a marked bacterial colonization in group II and a lower presence of bacteria in group III, despite no significant difference exists. It has been described that platelet aggregation together with fibrin and blood clots embeds bacteria at the site of infection withstanding the shear forces of blood flow [61]. The small difference between group II and group III with a clear decrease of bacterial count in group III can be explained by the antiplatelet activity of PGE_1_ that reduces the platelet entrapping of bacteria, thus the resistance of staphylococcal biofilm to antibiotic, as also demonstrated by others [61], [62]. This phenomenon is also supported by the histological results where the Gram positive staining highlighted a mild presence of dispersed bacteria within the medullary canal of group III, differently from bacterial clusters detected in group II. This effect, together with the increase of peripheral blood flow, contributes to a better host response to infection.

This study is effectively a single-dose study and the results are limited to this condition. Further evaluations with a larger group of animals are also necessary to investigate the immune system mechanisms, signaling pathways and the vascular changes as well as different PGE_1_ dosages and systemic implications. However, the present study provides some interesting observations that prove the positive effects of the synergic PGE_1_-antibiotic treatment on implant-related *S. aureus* orthopedic infections in diabetic mice.

## Conclusions

In conclusion, we have successfully visualized and quantified bacterial colonization in diabetic mice treated with the association of a cephalosporin and a PGE_1_ vasodilator. We were able to investigate the infectious processes throughout the course of the disease in the chronic phases comparing with an untreated sham control and with animals treated with a standard antibiotic therapy. To our knowledge, this is the first study describing the use of PGE_1_ as prophylaxis of the osteomyelitis and bacterial aggregation in diabetic implant-related bacterial infections. This novel approach, employing a validated diabetic animal model, can be used to examine in depth the signaling pathways and mechanisms activated by vasodilators to prevent osteomyelitis and to evaluate innovative therapeutic strategies for human use.
